# Duration-Dependent Aerobic Exercise Attenuates HFD-Induced MASLD in Association with Improved Hepatic Iron Homeostasis and Reduced Ferroptosis-Related Alterations

**DOI:** 10.3390/metabo16090626

**Published:** 2026-08-28

**Authors:** Lin Xu, Chang Li, Yufei Liu, Shijie Wang

**Affiliations:** 1 College of Exercise Science and Health, Harbin Sport University, Harbin 150008, China; 2 Graduate School, Harbin Sport University, Harbin 150008, China; 3 Laboratory Management Center, Harbin Sport University, Harbin 150008, China; liuyufei@hrbipe.edu.cn

**Keywords:** MASLD, aerobic exercise, exercise load, iron homeostasis, ferroptosis, hepcidin

## Abstract

**Highlights:**

**What are the main findings?**

- Moderate- and high-load aerobic exercise (especially the 90-min high-load regimen) significantly ameliorated HFD-induced metabolic abnormalities, hepatic steatosis, and liver injury in MASLD rats, with high-load exercise producing the most consistent improvements across most outcomes.
- Exercise interventions were accompanied by lower IL-6/JAK2/STAT3-hepcidin signaling, restored hepatic iron transport proteins (downregulated DMT1, upregulated FPN1), reduced Fe^2+^ accumulation, upregulated GPX4, downregulated PTGS2, and enhanced antioxidant capacity (increased GSH/SOD, decreased MDA), indicating attenuated ferroptosis-related alterations.

**What are the implications of the main findings?**

- These findings provide associative evidence that aerobic exercise-induced attenuation of MASLD is accompanied by improved hepatic iron homeostasis, reduced ferroptosis susceptibility, and lower IL-6/JAK2/STAT3–hepcidin signaling activity. However, the present study does not establish that this pathway mediates the effects of exercise.
- Duration-based aerobic exercise load (cumulative exercise volume) may influence the magnitude and consistency of hepatoprotection; however, as pathway-specific inhibitors or genetic models were not used, further mechanistic and dose–response studies are needed to establish causal relationships and define the optimal exercise prescription.

**Abstract:**

**Background/Objectives**: Aerobic exercise is widely recognized as an effective non-pharmacological strategy for metabolic dysfunction-associated steatotic liver disease (MASLD). However, the effects of different aerobic exercise loads on MASLD-associated hepatic iron dyshomeostasis and ferroptosis, as well as the underlying mechanisms, remain unclear. This study aimed to compare the effects of different aerobic exercise loads on HFD-induced MASLD and to explore whether exercise-induced improvements in hepatic lipid accumulation are associated with changes in iron homeostasis and ferroptosis-related markers. **Methods**: Forty male Sprague-Dawley rats were randomly assigned to a normal-fat diet group (NFD, *n* = 8) or a high-fat diet group (HFD, *n* = 32). After 8 weeks of HFD feeding, HFD-fed rats were further allocated to four groups: HFD control, low-load aerobic exercise (LEH), moderate-load aerobic exercise (MEH), and high-load aerobic exercise (HEH) (*n* = 8 per group). The exercise intervention lasted for 8 weeks. At the end of the intervention, blood and liver samples were collected to assess metabolic parameters, hepatic steatosis, iron homeostasis, oxidative stress, ferroptosis-related markers, and the IL-6/JAK2/STAT3-hepcidin pathway. **Results**: All three exercise regimens partially improved hepatic lipid metabolic abnormalities, iron accumulation, and oxidative stress-related liver injury in MASLD rats. These effects were accompanied by suppression of the IL-6/JAK2/STAT3-hepcidin pathway, increased hepatic FPN1 mRNA expression and decreased DMT1 mRNA expression, increased GPX4 expression, reduced PTGS2 expression, and improved antioxidant capacity. MEH and HEH generally produced larger changes than LEH, with HEH showing the most consistent overall response across the measured outcomes. **Conclusions**: Aerobic exercise attenuated HFD-induced MASLD in association with improved hepatic iron handling and a lower burden of ferroptosis-related molecular alterations. Because ferroptosis-specific rescue experiments and pathway inhibitors were not used, these findings are associative. Within the tested conditions, the 90-min protocol produced the most consistent response, but further dose–response studies are required before translation to humans.

## 1. Introduction

Metabolic dysfunction-associated steatotic liver disease (MASLD) is one of the most prevalent chronic liver diseases worldwide. Its defining feature is excessive hepatic fat accumulation, which is closely associated with metabolic risk factors such as obesity, insulin resistance, and type 2 diabetes [1]. As hepatic lipid accumulation progresses, insulin resistance (IR), oxidative stress, and lipotoxicity may aggravate hepatocellular injury and promote the transition from simple steatosis to metabolic dysfunction-associated steatohepatitis (MASH) [2]. Inflammation and oxidative stress are also key contributors to hepatic ferroptosis. Under pathological conditions such as obesity, diabetes, and MASLD, chronic inflammation can stimulate hepcidin synthesis through inflammatory cytokines such as interleukin-6 (IL-6), resulting in hepatic iron retention and increased susceptibility to ferroptosis [3,4]. In turn, hepatic iron overload further aggravates oxidative stress and cellular injury, thereby accelerating MASLD progression [5]. Thus, hepatic iron dyshomeostasis and ferroptosis may form a vicious cycle that promotes the progression of MASLD toward MASH and fibrosis. Although the molecular mechanisms of MASLD are increasingly understood, pharmacological treatment options remain limited [6]. Lifestyle intervention therefore remains a cornerstone of MASLD management. Exercise has been widely shown to improve hepatic steatosis, insulin sensitivity, and cardiometabolic function [6]. Previous studies suggest that exercise may reduce hepatic iron accumulation and ferroptosis-related changes by attenuating IL-6 signaling and lipid peroxidation, thereby modulating MASLD progression [7,8,9]. However, whether different aerobic exercise loads exert distinct effects on hepatic ferroptosis under MASLD conditions remains unclear. From the perspective of ferroptosis in MASLD progression, the present study aimed to compare the effects of different duration-based aerobic exercise loads on hepatic lipid metabolism, iron homeostasis, and ferroptosis-related alterations in HFD-induced MASLD rats, and to explore their association with the IL-6/JAK2/STAT3–hepcidin pathway.

## 2. Materials and Methods

### 2.1. Animals and Experimental Design

All animal experiments were conducted in accordance with the National Institutes of Health Guide for the Care and Use of Laboratory Animals and were approved by the Institutional Animal Care and Use Committee (IACUC) of Harbin Sport University on 9 April 2024 (Approval No. 2024038). Forty healthy male Sprague–Dawley rats, aged 6 weeks and weighing 200 ± 20 g, were obtained from Liaoning Changsheng Biotechnology Co., Ltd. (Shenyang, China). The animals were conventionally bred, non-genetically modified, and had not undergone any previous experimental procedures, pharmacological treatments, dietary interventions, or surgical manipulations before enrollment. Health status was verified using the supplier’s health certificate. No additional immune-status testing was performed specifically for this study. Rats were housed in standard polycarbonate rat cages measuring approximately 60 × 40 × 20 cm, with no more than six animals per cage. Animals assigned to different dietary or exercise groups were housed separately and were not mixed within the same cage. Autoclaved wood-shaving bedding was provided at a depth of approximately 2–3 cm and replaced twice weekly or earlier when visibly soiled or wet. Cages, food hoppers, and water bottles were cleaned and disinfected weekly using an institutionally approved disinfectant, thoroughly rinsed, and dried before reuse. Environmental enrichment included paper tubes and wooden gnawing blocks, which were inspected regularly and replaced when soiled or damaged. Animals were maintained at 21–25 °C and 40–50% relative humidity under a 12-h light/dark cycle, with ad libitum access to food and water. Bedding condition and cage cleanliness were checked daily.

**Figure 1:**
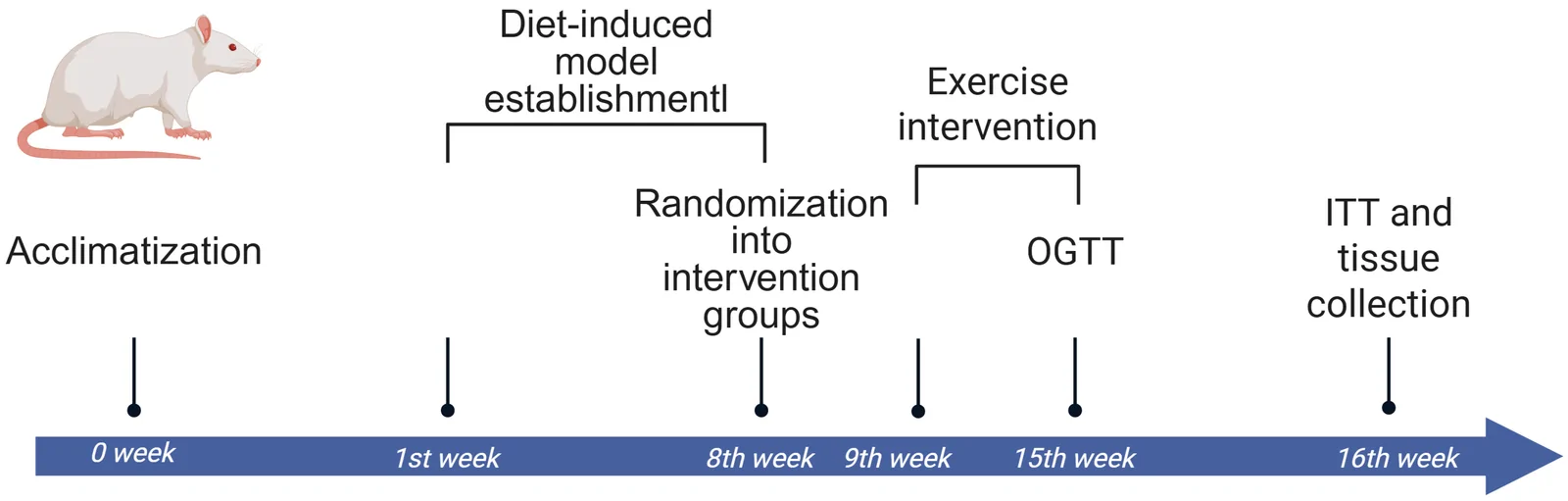
Experimental design and timeline.

After 1 week of acclimatization, rats were stratified according to baseline body weight and randomly allocated to two dietary groups using a computer-generated random-number sequence. The NFD group (*n* = 8) was fed a normal-fat diet (NFD, H10010, Beijing Huafukang Biotechnology Co., Ltd., Beijing, China) containing 10 kcal% fat, whereas the HFD group (*n* = 32) was fed a high-fat diet (HFD, H10060, Beijing Huafukang Biotechnology Co., Ltd., Beijing, China) containing 60 kcal% fat. The dietary intervention lasted for 8 weeks to induce hepatic steatosis. After 8 weeks of HFD feeding, the HFD-fed rats were stratified according to body weight and randomly allocated to the HFD, LEH, MEH, and HEH groups (*n* = 8 per group) using a computer-generated random-number sequence, thereby maintaining comparable body-weight distributions among the groups. Each individual rat was considered an independent experimental unit. No NFD-plus-exercise group was included because the primary objective was to compare three exercise durations as therapeutic interventions after HFD-induced MASLD had been established. Consequently, the design cannot distinguish responses specific to the MASLD state from exercise effects that might also occur in healthy animals; this limitation is acknowledged below. The overall experimental design and timeline are shown in Figure 1.

### 2.2. Sample Size Determination

No formal a priori sample-size calculation was performed. The group size of eight animals was selected based on sample sizes commonly used in comparable HFD-induced MASLD and treadmill-exercise studies, the expected biological variability of the measured outcomes, experimental feasibility, and the principle of reducing animal use. Accordingly, eight rats were allocated to each of the five final experimental groups, resulting in a total sample size of 40 animals. Assay-specific sample sizes were determined according to the availability of biological material and the planned assay capacity and are reported in the corresponding figure legends. The qRT-PCR and Western blot analyses were limited to three independent biological replicates per group and should therefore be regarded as exploratory. The study was not powered to provide definitive molecular-effect estimates.

To minimize potential confounding, all animals were housed under the same environmental conditions, and feeding, handling, exercise training, body-weight measurements, glucose testing, and sample collection were performed according to standardized procedures and within consistent daily time windows. The same trained personnel conducted the exercise interventions and sample collection. Blinding was not applied during group allocation or implementation of the dietary and treadmill exercise interventions because the assigned interventions were readily distinguishable. Personnel conducting the biochemical assays, qRT-PCR, Western blotting, and statistical analyses were not formally blinded to group allocation. Histological assessment was performed under blinded conditions as described below.

### 2.3. Inclusion and Exclusion Criteria and Selection of Biological Replicates

Eligibility and exclusion criteria were defined before outcome analysis. Animals were eligible for inclusion if they remained clinically healthy after acclimatization and completed the assigned dietary and exercise intervention. Animals were to be excluded if they died during the experiment, developed an illness or injury unrelated to the intervention that could substantially affect the study outcomes, or were unable to complete the prescribed intervention. Individual outcome data were excluded only in cases of documented technical failure, such as insufficient or severely haemolyzed serum, inadequate tissue quantity, sample degradation, or assay failure. Data were not excluded solely on the basis of their magnitude, statistical significance, or inconsistency with the study hypothesis.

All 40 animals completed the experimental protocol and were included in the study. All eight animals in each group were included in the analyses of body weight and liver weight. For analyses reported with six biological replicates per group, six animals were selected from the eight animals in each group by simple random sampling without replacement using a computer-generated random-number sequence before outcome measurement. For qRT-PCR and Western blot analyses reported with three biological replicates per group, three animals were independently selected from each group using the same procedure. Sample selection was based exclusively on coded animal identification numbers and was independent of treatment response or measured outcome values. Each animal was considered one independent biological replicate. Technical replicates obtained from the same animal were averaged and were not treated as independent observations. The exact sample size for each analysis is reported in the corresponding figure legend. For the molecular assays, *n* = 3 denotes three independently selected animals and not technical replicates.

Rats in the exercise groups performed treadmill training using a ZS-PT-III treadmill (Zhongshi Dicreat Technology Development Co., Ltd., Beijing, China). The three exercise protocols differed mainly in training duration and were performed for 8 weeks. The training protocols were adapted from Deng et al. [10] with minor modifications, as shown in Table 1. Body weight was recorded at the same time of day once weekly throughout both the 8-week model-establishment period and the 8-week exercise-intervention period. Absolute body weight (g) was used for the longitudinal analysis. Percentage body-weight change was calculated for each rat separately for the model-establishment and exercise-intervention periods as [(week 8 body weight − week 0 body weight)/week 0 body weight] × 100% and [(week 16 body weight − week 8 body weight)/week 8 body weight] × 100%, respectively; group means were then summarized descriptively.

### 2.4. Animal Monitoring and Humane Endpoints

Animals were monitored at least once daily throughout the study by trained personnel. General health assessments included appearance, posture, spontaneous activity, gait, respiration, grooming, food and water intake, defecation, hydration status, and the presence of visible injury or abnormal behavior. During treadmill adaptation and exercise sessions, animals were examined before training, continuously observed during exercise, and reassessed immediately after each session for abnormal gait, excessive fatigue, respiratory distress, injury, or inability to continue running.

To minimize distress, animals underwent a gradual treadmill adaptation program before the formal exercise intervention. Handling and sample collection were performed using gentle restraint. Exercise was immediately discontinued if an animal showed marked respiratory distress, persistent abnormal gait, injury, inability to maintain a normal posture, or severe exhaustion. After each exercise session, animals were returned to their home cages and allowed free access to food and water.

Humane endpoints were predefined before the experiment. Animals were to be removed from the study and evaluated if they exhibited persistent inability to obtain food or water, severe or persistent lethargy, loss of the righting reflex, labored breathing, severe dehydration, uncontrolled bleeding or injury, persistent inability to stand or walk normally, or a sustained loss of more than 20% of body weight relative to the most recent stable measurement. Animals unlikely to recover or experiencing distress that could not be promptly alleviated were to be humanely euthanized in accordance with the protocol approved by the institutional animal ethics committee.

No animal reached a predefined humane endpoint, and no unexpected adverse events occurred during the study. Transient fatigue observed immediately after treadmill exercise resolved during the post-exercise recovery period without additional intervention.

At the end of the experiment, rats were fasted overnight and deeply anesthetized with ether. Adequate anesthetic depth was confirmed before laparotomy and terminal blood collection. The abdominal aorta was exposed and blood was collected directly during terminal exsanguination while the animals remained under deep anesthesia; no blood was collected from conscious animals. Death was confirmed before liver removal. Serum was separated by centrifugation. Liver tissues were either fixed in 4% paraformaldehyde for morphological assessment or snap-frozen in liquid nitrogen and stored at −80 °C for subsequent analyses.

### 2.5. Glucose Tolerance and Insulin Resistance Analysis

In the seventh and eighth weeks of the intervention, rats underwent an oral glucose tolerance test (OGTT) and an insulin tolerance test (ITT), respectively. For the OGTT, rats were fasted for 12 h and then administered glucose (2 g/kg body weight) by oral gavage. Blood glucose levels were measured from tail vein blood at 0, 30, 60, 90, and 120 min after glucose administration. The area under the curve (AUC) was calculated over the 120-min period. For the ITT, rats were fasted for 6 h and then intraperitoneally injected with insulin (0.75 IU/kg). Blood glucose was monitored at the same time points as those used for the OGTT. All groups, including the NFD and non-exercised HFD control groups, underwent identical fasting, handling, tail-vein sampling, and oral glucose-gavage procedures. A placebo-only group was not included because the OGTT is defined by a standardized glucose challenge; thus, potential stress associated with handling and gavage was applied equally across groups.

### 2.6. Blood Biochemical Analysis

Serum levels of total cholesterol (TC), triglycerides (TG), alanine aminotransferase (ALT), aspartate aminotransferase (AST), low-density lipoprotein cholesterol (LDL-C), and high-density lipoprotein cholesterol (HDL-C) were quantified using an automated biochemical analyzer (BS-2000M, Shenzhen Mindray Bio-Medical Electronics Co., Ltd., Shenzhen, China).

### 2.7. Histological Staining

Liver tissues were fixed in 4% paraformaldehyde for 24 h, dehydrated, embedded in paraffin, and sectioned. Sections were stained with hematoxylin and eosin (H&E) for histopathological examination. Steatosis (0–3), lobular inflammation (0–3), and hepatocyte ballooning (0–2) were scored according to the criteria described by Kleiner et al. [11], with a total score ranging from 0 to 8. Scoring was performed independently by two pathologists blinded to the experimental groups, and any disagreement was resolved by a third pathologist. For Oil Red O staining, liver samples were embedded, cryosectioned, and stained according to standard protocols. Hepatic iron deposition was assessed using a Prussian blue iron staining kit (Servicebio, Wuhan, China). Oil Red O- and Prussian blue-stained images were analyzed using ImageJ v1.53 (National Institutes of Health, Bethesda, MD, USA), and the mean value from three fields of view per sample was used for quantitative analysis. Histological images were captured using a Moticam 3000 microcamera imaging system (Motic China Group Co., Ltd., Xiamen, China).

### 2.8. Tissue Ferrous Ion Measurement

Ferrous ion (Fe^2+^) concentrations in liver tissues were determined using a commercial ferrous ion assay kit (Suzhou Grise Biotechnology Co., Ltd., Suzhou, China) according to the manufacturer’s instructions. The results were normalized according to the assay protocol.

### 2.9. Measurement of Ferroptosis Markers

Ferroptosis- and oxidative stress-related markers were measured using commercial assay kits. Malondialdehyde (MDA) levels were determined using a lipid peroxidation assay kit (Suzhou Gerace Biotechnology Co., Ltd., Suzhou, China), whereas glutathione (GSH) and superoxide dismutase (SOD) levels were measured using enzyme-linked immunosorbent assay kits (Shanghai Enzyme Link Technology Co., Ltd., Shanghai, China). Liver tissues were homogenized in phosphate-buffered saline (PBS) and centrifuged to obtain supernatants. Protein concentrations were determined using the bicinchoninic acid (BCA) assay and used for normalization.

### 2.10. Quantitative Real-Time PCR Analysis

Total RNA was extracted from liver tissues using TRIzol Up (Beijing AllStyle Gold Bioscience & Technology Co., Ltd., Beijing, China) and reverse-transcribed into cDNA using the EasyScript One-Step gDNA Removal and cDNA Synthesis SuperMix kit (Beijing AllStyle Gold Bioscience & Technology Co., Ltd., Beijing, China). Quantitative real-time PCR (qRT-PCR) was performed using an FQD-96A RT-PCR system (Hangzhou BORI Technology Co., Ltd., Hangzhou, China) and TransStart Top Green qPCR SuperMix (Beijing TransGen Biotech Co., Ltd., Beijing, China). Primer sequences are listed in Table 2. Gene expression was normalized to beta-actin and calculated using the 2^−ΔΔCt^ method.

### 2.11. Western Blot Assay

Protein samples were separated by sodium dodecyl sulfate-polyacrylamide gel electrophoresis (SDS-PAGE) and transferred onto polyvinylidene difluoride (PVDF) membranes. Membranes were blocked with 5% skimmed milk for 2 h at room temperature and then incubated overnight at 4 °C with primary antibodies against IL-6 (1:1000, GB11117), STAT3 (1:1000, GB11176), p-STAT3 (1:1000, GB150001), p-JAK2 (1:1000, GB114585), JAK2 (1:1000, GB11325), GPX4 (1:1000, GB113745), PTGS2 (1:1000, GB115672), and beta-actin (1:1000, GB15003), all purchased from Servicebio (Wuhan, China). After four washes with TBST, membranes were incubated with HRP-conjugated secondary antibodies for 2 h at room temperature. Protein bands were visualized using an enhanced chemiluminescence detection system (GE HealthCare, Chicago, IL, USA).

### 2.12. Statistical Analysis

Data were analyzed using GraphPad Prism 9.5 (GraphPad Software, San Diego, CA, USA) and are presented as the mean ± standard error of the mean (SEM). Weekly body-weight trajectories and the serial blood-glucose values obtained during the OGTT and ITT were analyzed using two-way repeated-measures ANOVA, with experimental group as the between-subject factor and time as the within-subject factor, followed by Tukey’s multiple-comparisons test. When the sphericity assumption was not met, the Geisser-Greenhouse correction was applied. Single-time-point outcomes, including liver weight, serum lipid concentrations, and OGTT/ITT area under the curve (AUC), were analyzed using one-way ANOVA followed by Tukey’s post hoc test. Statistical differences are expressed as follows: * *p* < 0.05; ** *p* < 0.01; *** *p* < 0.001; ns, not statistically significant. Owing to the small molecular sample size (*n* = 3 per group), qRT-PCR and Western blot comparisons were treated as exploratory and interpreted in conjunction with the biochemical and histological findings rather than as stand-alone mechanistic proof.

## 3. Results

### 3.1. Different Exercise Loads Ameliorate Metabolic Abnormalities in HFD-Induced MASLD Rats

**Figure 2:**
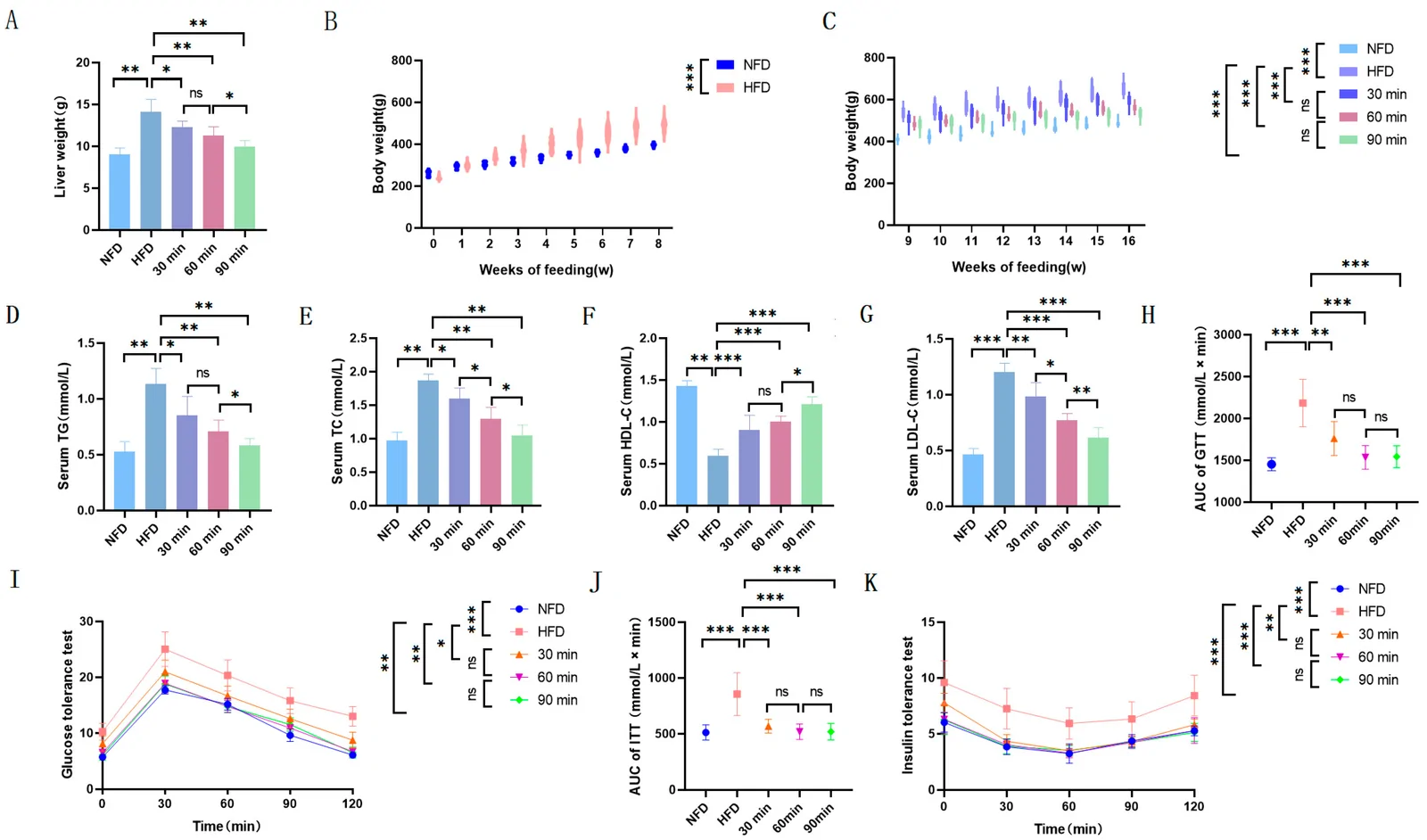
Different duration-based aerobic exercise loads ameliorate metabolic abnormalities in HFD-induced MASLD rats. (**A**) Liver weight at the end of the intervention (*n* = 8 per group). (**B**) Weekly body weight during the dietary model-establishment period (weeks 0–8; NFD, *n* = 8; HFD, *n* = 32 before secondary randomization). (**C**) Weekly body weight during the exercise-intervention period (weeks 9–16; *n* = 8 per group). (**D**–**G**) Serum TG, TC, HDL-C, and LDL-C concentrations, respectively (*n* = 6 per group). (**H**) AUC for the oral glucose tolerance test (OGTT) performed in week 7 of the exercise intervention (*n* = 6 per group). (**I**) OGTT blood-glucose response at 0, 30, 60, 90, and 120 min. (**J**) AUC for the insulin tolerance test (ITT) performed in week 8 of the exercise intervention (*n* = 6 per group). (**K**) ITT blood-glucose response at 0, 30, 60, 90, and 120 min. Weekly body-weight trajectories and OGTT/ITT time-course data were analyzed by two-way repeated-measures ANOVA (group x time), followed by Tukey’s multiple-comparisons test. Liver weight, serum lipid concentrations, and AUC values were analyzed by one-way ANOVA followed by Tukey’s post hoc test. Data are presented as mean ± SEM. * *p* < 0.05; ** *p* < 0.01; *** *p* < 0.001; ns, not statistically significant.

As shown in Figure 2B, body weight increased progressively in both the NFD and HFD groups during the 8-week model-establishment period. From week 0 to week 8, body weight increased by 52.50% in the NFD group and by 110.40% in the HFD group, indicating a substantially greater body-weight gain after HFD feeding. During the subsequent 8-week exercise-intervention period, body weight continued to increase in all groups (Figure 2C). Relative to body weight at week 8, the increases at week 16 were 19.07%, 23.01%, 16.91%, 15.83%, and 10.39% in the NFD, HFD, LEH, MEH, and HEH groups, respectively. The magnitude of body-weight gain was therefore descriptively lower in the exercise groups than in the HFD group, with the smallest increase observed in the HEH group. At the end of the intervention, liver weight was significantly higher in the HFD group than in the NFD group, whereas exercise reduced liver weight to varying degrees, with greater reductions observed in the MEH and HEH groups (Figure 2A).

Serum lipid analysis showed that HFD-fed rats had significantly higher TG, TC, and LDL-C concentrations and lower HDL-C concentrations than NFD-fed rats (*p* < 0.05), confirming HFD-induced dyslipidemia. All three exercise regimens reduced TG, TC, and LDL-C and increased HDL-C relative to the sedentary HFD group (*p* < 0.05). For TG and HDL-C, the 30- and 60-min groups did not differ significantly, whereas the 90-min group showed a further improvement relative to the 60-min group (*p* < 0.05). TC and LDL-C improved progressively across the 30-, 60-, and 90-min groups, with significant differences between adjacent exercise-duration groups (*p* < 0.05; Figure 2D–G).

The OGTT and ITT time-course analyses showed that HFD feeding impaired glucose tolerance and insulin sensitivity, as indicated by higher blood-glucose responses and larger AUC values than those in the NFD group (*p* < 0.05; Figure 2H–K). Each exercise regimen significantly reduced the OGTT and ITT responses and their corresponding AUCs relative to the HFD group (*p* < 0.05). However, the AUC values did not differ significantly between the 30- and 60-min groups or between the 60- and 90-min groups (*p* > 0.05). Thus, all three exercise durations improved glucose tolerance and insulin sensitivity, but these endpoints did not show a clear duration-dependent gradient within the tested range.

### 3.2. Different Exercise Loads Attenuate Hepatic Steatosis and Liver Injury in MASLD Rats

**Figure 3:**
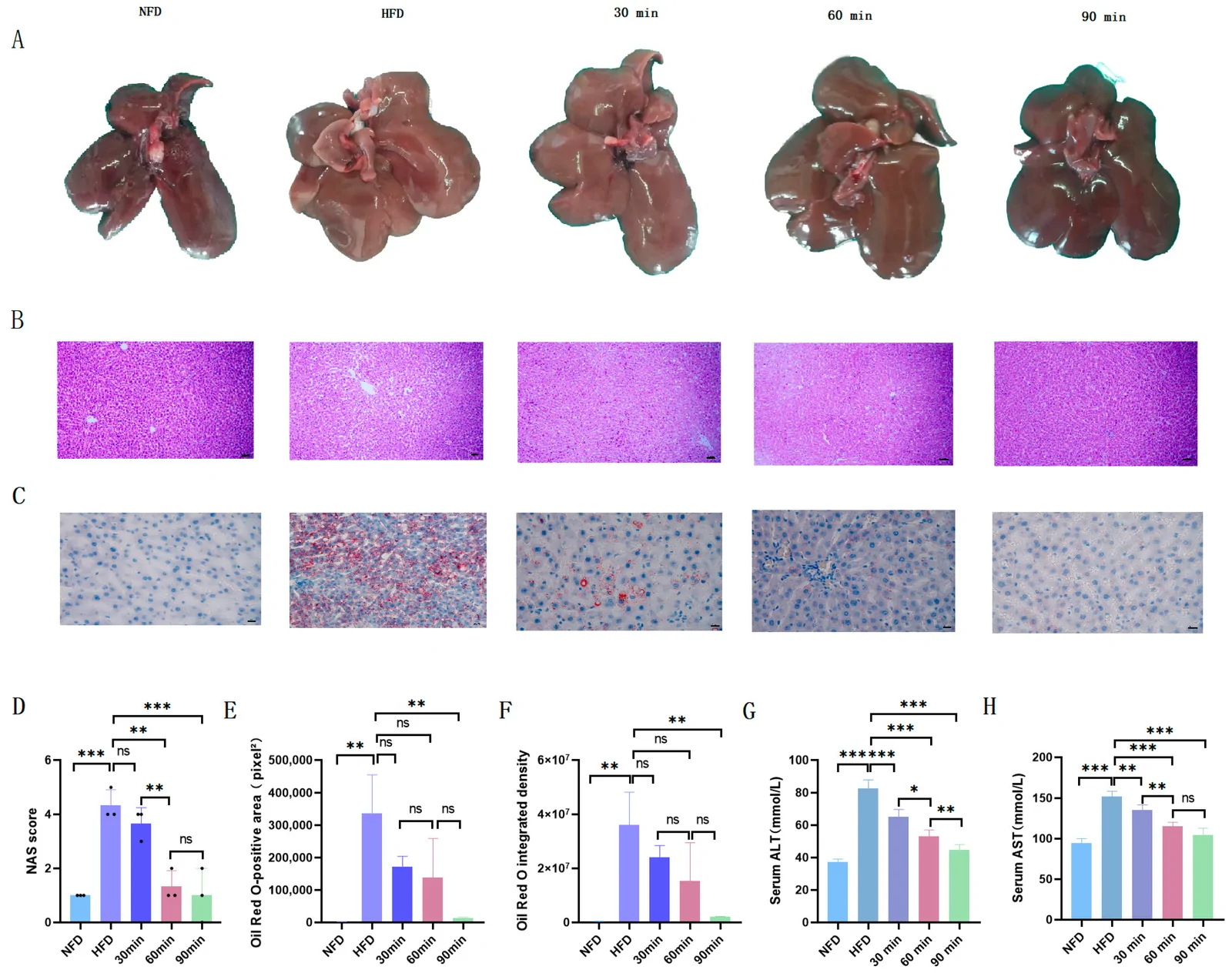
Different exercise loads attenuate hepatic steatosis and liver injury in MASLD rats. (**A**) Liver image. (**B**) H&E staining (scale bar: 200 μm). (**C**) Oil Red O staining (scale bar: 200 μm). (**D**) NAS score. (**E**) Total area of Oil Red O-positive regions. (**F**) Integrated density of positive regions. (**G**) Serum ALT (*n* = 6). (**H**) Serum AST (*n* = 6). The results are presented as the mean ± SEM. Differences were assessed by one-way ANOVA. * *p* < 0.05; ** *p* < 0.01; *** *p* < 0.001; ns for not statistically significant.

As shown in Figure 3, livers from HFD-fed rats were enlarged and pale, exhibiting typical features of hepatic steatosis. Exercise intervention visibly improved liver appearance, particularly in the MEH and HEH groups, in which liver color and morphology were closer to those observed in the NFD group (Figure 3A).

H&E staining showed that the HFD group exhibited prominent lipid vacuoles, disorganized hepatocyte arrangement, and structural injury in liver tissue. These pathological alterations were alleviated after exercise intervention. NAS scoring further showed that the HFD group had a significantly higher score than the NFD group (*p* < 0.05), confirming successful establishment of the MASLD model. NAS scores were significantly reduced in the MEH and HEH groups (*p* < 0.05), with MEH showing a greater decrease than LEH and HEH showing a further improving trend (Figure 3B,D).

Oil Red O staining revealed marked hepatic lipid droplet accumulation in the HFD group, accompanied by significant increases in Oil Red O-positive area and integrated optical density (*p* < 0.05). Exercise reduced hepatic lipid deposition. In particular, HEH significantly decreased both the Oil Red O-positive area and integrated optical density (*p* < 0.05), suggesting that a higher exercise load exerted a stronger effect on reducing hepatic lipid accumulation (Figure 3C,E,F).

Serum liver function analysis showed that ALT and AST levels were significantly elevated in the HFD group (*p* < 0.05), indicating liver injury in MASLD rats. Exercise significantly reduced ALT and AST levels (*p* < 0.05). MEH decreased ALT more effectively than LEH, while HEH further reduced ALT compared with MEH (*p* < 0.05) (Figure 3G,H).

### 3.3. Different Exercise Loads Improve Hepatic Iron Homeostasis in MASLD Rats

**Figure 4:**
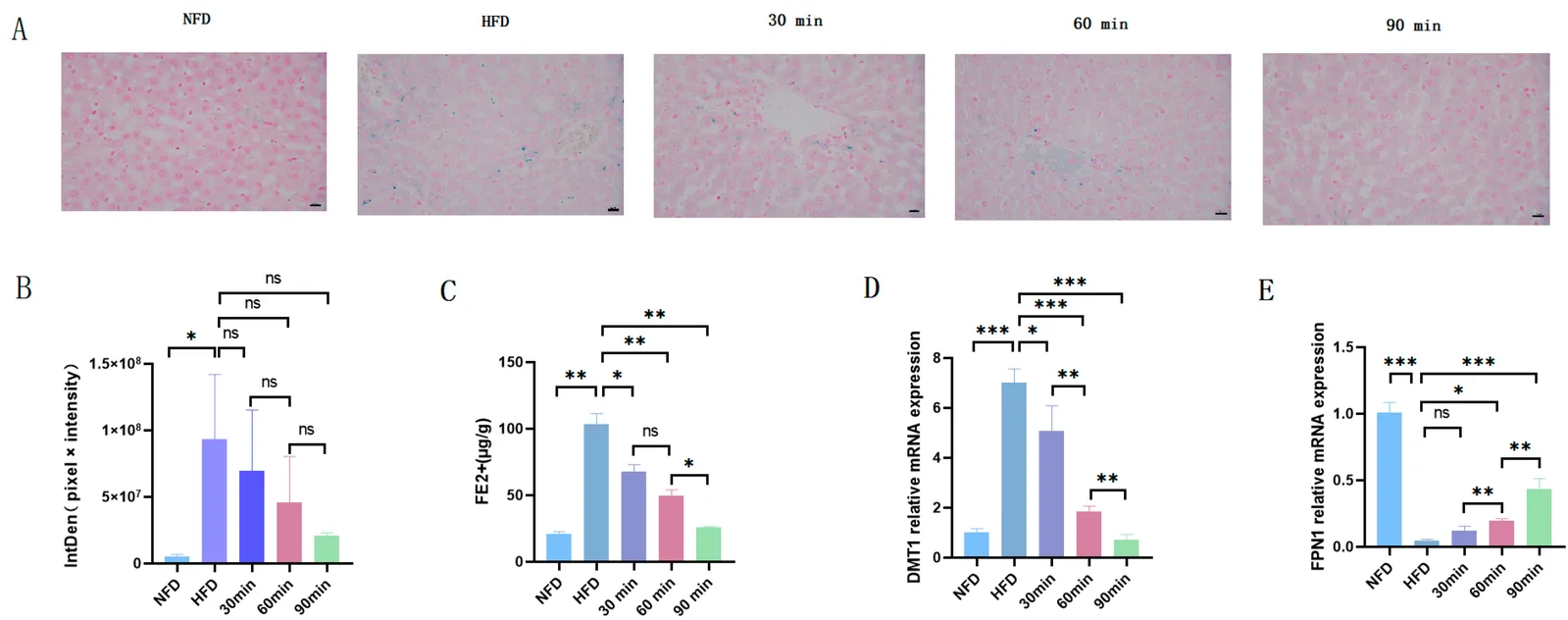
Different aerobic exercise loads alleviate hepatic iron dyshomeostasis in MASLD rats. (**A**) Prussian blue iron staining (scale: 200 μm). (**B**) Integrated optical density of Prussian blue-positive regions. (**C**) Ferrous ion content. (**D**,**E**) Relative mRNA levels of DMT1, FPN1 in the liver (*n* = 3). The results are presented as the mean ± SEM. Differences were assessed by one-way ANOVA. * *p* < 0.05; ** *p* < 0.01; *** *p* < 0.001; ns for not statistically significant. The qRT-PCR data represent *n* = 3 independent biological replicates per group and are exploratory.

To evaluate the effects of exercise on hepatic iron metabolism in MASLD rats, hepatic iron deposition, Fe^2+^ content, and the expression of iron transport-related molecules were measured. As shown in Figure 4A, Prussian blue staining revealed obvious hepatic iron deposition in the HFD group, whereas iron deposition tended to decrease after exercise intervention. Quantitative analysis showed that the cumulative optical density of Prussian blue-positive areas was significantly higher in the HFD group than in the NFD group (*p* < 0.05), indicating HFD-induced hepatic iron accumulation. After exercise intervention, the cumulative optical density tended to decrease, although the difference did not reach statistical significance (*p* > 0.05) (Figure 4B).

Hepatic Fe^2+^ levels were significantly elevated in the HFD group (*p* < 0.05), indicating hepatic iron overload under MASLD conditions. Compared with the HFD group, LEH, MEH, and HEH significantly reduced hepatic Fe^2+^ levels (*p* < 0.05). MEH produced a greater reduction than LEH, and HEH showed the most pronounced decrease (*p* < 0.05) (Figure 4C).

At the molecular level, DMT1 mRNA expression was significantly increased, whereas FPN1 mRNA expression was significantly decreased in the HFD group (*p* < 0.05), indicating enhanced hepatic iron uptake and impaired iron export in MASLD rats. Exercise markedly decreased DMT1 expression and increased FPN1 expression. In the LEH group, the increase in FPN1 did not reach statistical significance (*p* > 0.05), whereas MEH and HEH significantly restored FPN1 expression (*p* < 0.05). Intergroup comparisons showed that HEH exerted stronger regulatory effects on DMT1 and FPN1 than MEH, and MEH was more effective than LEH (*p* < 0.05) (Figure 4D,E).

### 3.4. Different Exercise Loads Are Associated with Suppression of the IL-6/JAK2/STAT3–Hepcidin Signaling Pathway

**Figure 5:**
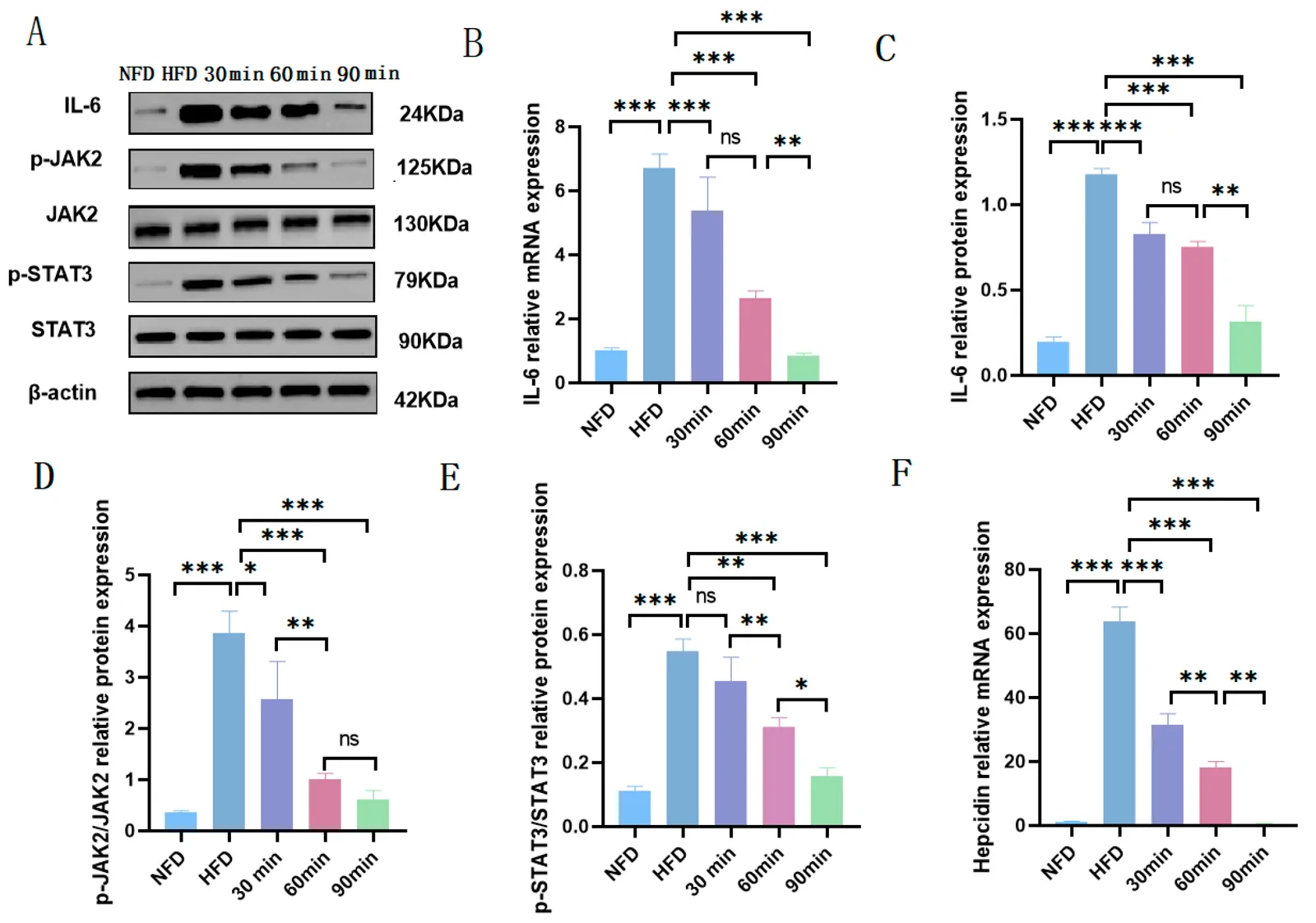
Different aerobic exercise loads are associated with reduced IL-6/JAK2/STAT3–hepcidin signaling in HFD-induced MASLD rats. (**A**) Protein bands. (**B**) IL-6 mRNA levels. (**C**) IL-6 protein expression analysis. (**D**) Phosphorylation of JAK2. (**E**) Phosphorylation of STAT3. (**F**) Hepatic hepcidin mRNA levels. The results are presented as the mean ± SEM. Differences were assessed by one-way ANOVA. * *p* < 0.05; ** *p* < 0.01; *** *p* < 0.001; ns for not statistically significant. Molecular analyses were performed using *n* = 3 independent biological replicates per group and should be interpreted as exploratory.

To investigate the potential mechanism by which exercise improves hepatic iron homeostasis, the IL-6/JAK2/STAT3-hepcidin signaling pathway was examined. As shown in Figure 5, both hepatic IL-6 mRNA and protein expression were significantly increased in the HFD group compared with the NFD group (*p* < 0.05), indicating enhanced hepatic inflammation under MASLD conditions. Exercise markedly reduced IL-6 expression, with the MEH and HEH groups showing more pronounced decreases (*p* < 0.05). Compared with LEH, MEH exerted a stronger inhibitory effect on IL-6 mRNA, whereas HEH further decreased IL-6 protein expression compared with MEH (*p* < 0.05) (Figure 5A–C).

Western blot analysis showed that p-JAK2/JAK2 and p-STAT3/STAT3 levels were significantly increased in the HFD group (*p* < 0.05), indicating activation of JAK2/STAT3 signaling by HFD feeding. Exercise markedly reduced JAK2 and STAT3 phosphorylation. All three exercise loads significantly decreased p-JAK2/JAK2 levels (*p* < 0.05), with MEH being more effective than LEH. For p-STAT3/STAT3, LEH showed a downward trend that did not reach statistical significance (*p* > 0.05), whereas both MEH and HEH significantly reduced this marker (*p* < 0.05). HEH was more effective than MEH, and MEH was more effective than LEH (*p* < 0.05) (Figure 5D,E).

In addition, hepcidin mRNA expression was significantly elevated in the HFD group (*p* < 0.05), suggesting that HFD feeding promotes hepcidin transcription through inflammation-related signaling. Exercise significantly decreased hepcidin mRNA expression, with the greatest reduction observed in the HEH group, followed by MEH and LEH (*p* < 0.05) (Figure 5F).

Together, these results suggest that different aerobic exercise loads may improve hepatic iron metabolism in association with suppression of the IL-6/JAK2/STAT3–hepcidin axis.

### 3.5. Different Exercise Loads Attenuate Hepatic Ferroptosis-Related Alterations in MASLD Rats

**Figure 6:**
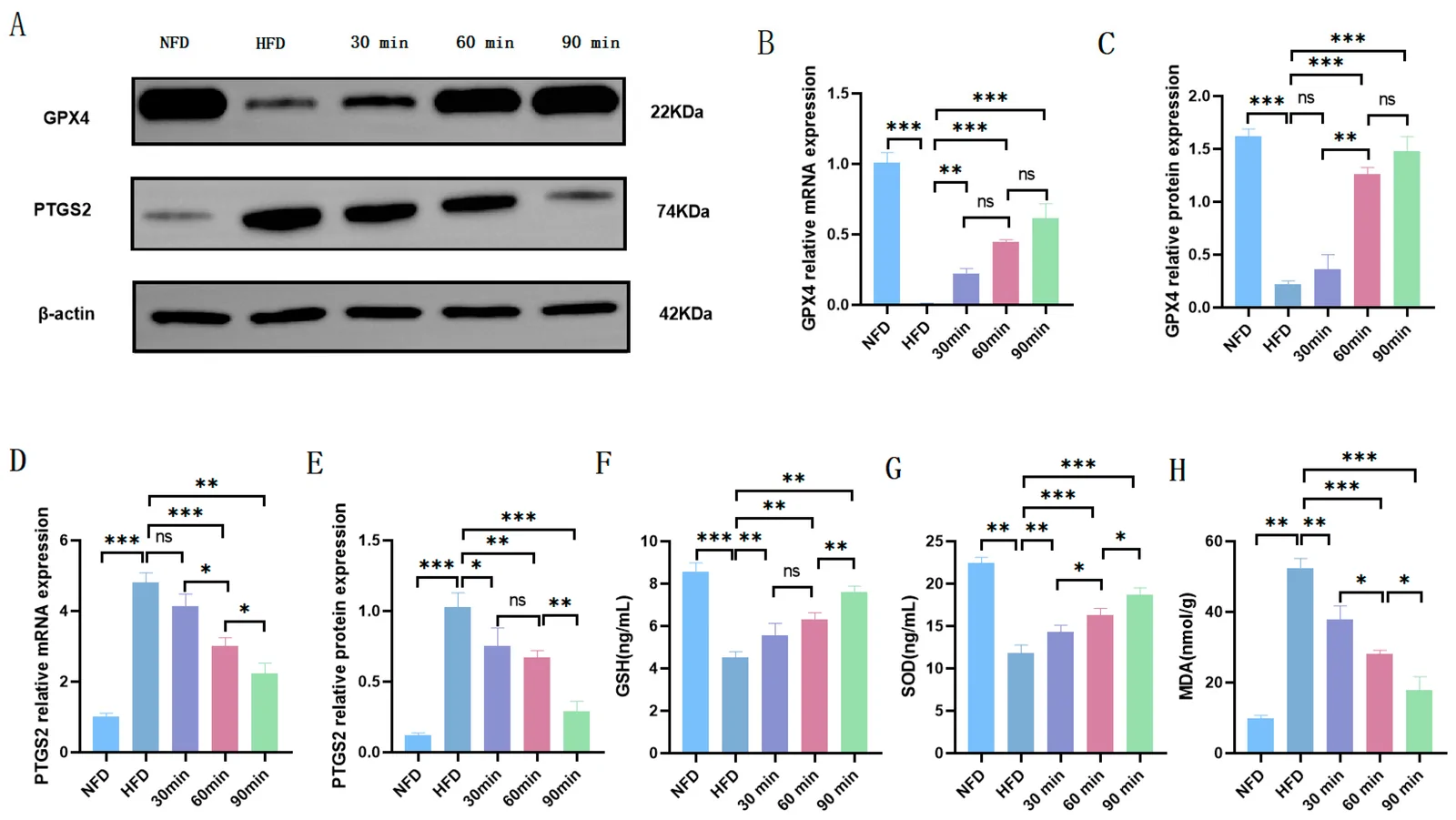
Different exercise loads are associated with attenuation of hepatic ferroptosis-related molecular alterations in MASLD rats. (**A**) Representative Western blot bands. (**B**,**C**) Relative hepatic GPX4 mRNA and protein expression, respectively (*n* = 3 independent biological replicates per group). (**D**,**E**) Relative hepatic PTGS2 mRNA and protein expression, respectively (*n* = 3 independent biological replicates per group). (**F**–**H**) Hepatic GSH, SOD, and MDA levels, respectively (*n* = 6 per group). Data are presented as mean ± SEM. Differences were assessed by one-way ANOVA followed by Tukey’s post hoc test. * *p* < 0.05; ** *p* < 0.01; *** *p* < 0.001; ns, not statistically significant.

To evaluate whether exercise was associated with changes in ferroptosis-related molecular features, GPX4, PTGS2, and oxidative-stress indicators were measured. As shown in Figure 6, GPX4 mRNA and protein expression were lower, whereas PTGS2 mRNA and protein expression were higher, in the HFD group than in the NFD group (*p* < 0.05). This pattern is consistent with impaired antioxidant defense and increased susceptibility to iron-dependent lipid peroxidation, but it does not by itself demonstrate ferroptotic cell death. Exercise increased GPX4 expression in all three intervention groups, with a stronger protein response in MEH than LEH and a further increasing trend in HEH (*p* < 0.05) (Figure 6A–C).

Concurrently, exercise reduced PTGS2 expression. Although LEH did not significantly decrease PTGS2 mRNA levels (*p* > 0.05), both MEH and HEH significantly reduced PTGS2 mRNA expression (*p* < 0.05). PTGS2 protein levels were significantly decreased after all three exercise interventions, with HEH showing a stronger effect than MEH and MEH showing a stronger effect than LEH (*p* < 0.05) (Figure 6D,E).

Oxidative stress analysis showed that GSH and SOD levels were significantly decreased, whereas MDA levels were significantly increased in the HFD group (*p* < 0.05), indicating impaired hepatic antioxidant capacity and enhanced lipid peroxidation in MASLD rats. Exercise significantly increased GSH and SOD levels and decreased MDA levels (*p* < 0.05). HEH produced more pronounced improvements in GSH, SOD, and MDA than MEH, whereas MEH was more effective than LEH in increasing SOD and reducing MDA (*p* < 0.05) (Figure 6F–H).

Collectively, the exercise groups showed partial reversal of ferroptosis-related molecular and oxidative-stress alterations, including higher GPX4, lower PTGS2, improved antioxidant capacity, and reduced lipid peroxidation. MEH and HEH generally produced larger changes than LEH, and HEH showed the most consistent profile. These surrogate outcomes support reduced ferroptosis susceptibility but do not establish direct inhibition of ferroptotic cell death.

## 4. Discussion

The present study compared three duration-based aerobic exercise protocols in HFD-induced MASLD rats and yielded four principal findings. First, aerobic exercise improved systemic metabolic abnormalities, hepatic steatosis, and liver injury, with the HEH protocol producing the most consistent changes across the measured outcomes. Second, these improvements were accompanied by reduced hepatic Fe^2+^ levels and partial normalization of the mRNA expression of the iron transport-related genes DMT1 and FPN1. Third, exercise-induced improvements in hepatic iron homeostasis occurred in parallel with reduced hepatic IL-6 expression, JAK2/STAT3 phosphorylation, and hepcidin mRNA expression. Fourth, exercise increased GPX4 expression and antioxidant capacity while reducing PTGS2 expression and lipid peroxidation. Collectively, these findings indicate that aerobic exercise attenuates HFD-induced MASLD in association with improved hepatic iron homeostasis and reduced ferroptosis-related alterations. However, because no pathway-specific or ferroptosis-specific interventions were used, the findings should be interpreted as associative rather than causal. In addition, the qRT-PCR and Western blot findings were based on *n* = 3 independent biological replicates per group and should be interpreted as exploratory.

### 4.1. Aerobic Exercise Partially Improves Hepatic Iron Homeostasis in HFD-Induced MASLD

A major finding of the present study was that HFD feeding increased hepatic Fe^2+^ levels, upregulated DMT1 mRNA expression, and downregulated FPN1 mRNA expression. This pattern is consistent with increased iron uptake and impaired cellular iron export under MASLD conditions. DMT1 contributes to cellular ferrous iron uptake, whereas FPN1 is the principal cellular iron exporter and is essential for the mobilization of hepatic and systemic iron stores [12,13,14,15]. Hepcidin binds to FPN1 and promotes its internalization and degradation, thereby restricting cellular iron export and favoring intracellular iron retention [3,12]. Therefore, the concurrent increase in hepcidin and DMT1 expression and decrease in FPN1 expression observed in the HFD group may favor hepatic iron accumulation.

Exercise partially reversed these alterations. All three exercise protocols reduced hepatic Fe^2+^ content, whereas MEH and HEH significantly increased FPN1 mRNA expression and decreased DMT1 mRNA expression. HEH produced the most pronounced changes, suggesting that a greater duration-based exercise load may provide stronger regulation of hepatic iron handling. Exercise has previously been proposed to influence iron homeostasis and ferroptosis through coordinated effects on iron transport, oxidative stress, and metabolic regulation [16,17].

Nevertheless, the present findings do not demonstrate complete restoration of hepatic iron homeostasis. Although Prussian blue staining showed a decreasing trend after exercise, the differences between the exercise groups and the HFD group did not reach statistical significance. In addition, DMT1 and FPN1 were assessed only at the mRNA level, and circulating iron, ferritin, transferrin saturation, and hepatic iron-storage proteins were not measured. The present results therefore support partial improvement in iron transport-related gene expression and the hepatic Fe^2+^ pool rather than definitive normalization of whole-body or hepatic iron metabolism.

The reduction in hepatic Fe^2+^ may nevertheless be biologically relevant because excessive iron promotes reactive oxygen species generation, lipid peroxidation, and ferroptosis-related cellular injury [7,17,18]. Iron-dependent lipid peroxidation is considered an important contributor to hepatocellular injury and MASLD progression [4,5,17]. Accordingly, the exercise-induced reduction in Fe^2+^ occurred together with lower MDA and PTGS2 levels and higher GPX4, GSH, and SOD levels. This concordant pattern supports a potential relationship between improved iron handling and reduced ferroptosis susceptibility, although it does not establish that changes in iron transport directly caused the improvements in hepatic pathology.

### 4.2. Reduced IL-6/JAK2/STAT3–Hepcidin Signaling Is Associated with Improved Iron Homeostasis

The HFD group exhibited increased hepatic IL-6 expression, JAK2 and STAT3 phosphorylation, and hepcidin mRNA expression. These changes were accompanied by increased hepatic Fe^2+^ and DMT1 expression and decreased FPN1 expression. IL-6-dependent activation of JAK/STAT3 signaling is an established stimulus for hepatic hepcidin transcription, whereas increased hepcidin restricts FPN1-mediated cellular iron export [3,19,20]. Studies in fatty liver and obesity-related models have also linked hepatic inflammation, altered hepcidin signaling, and iron accumulation [19,21]. The parallel alterations observed in the present study are therefore consistent with activation of an inflammation-associated iron-retention response in HFD-induced MASLD.

Exercise reduced hepatic IL-6 expression, p-JAK2/JAK2, p-STAT3/STAT3, and hepcidin mRNA expression, with MEH and HEH generally producing stronger changes than LEH. The exercise-induced reduction in hepatic IL-6 was accompanied by decreased JAK2/STAT3 phosphorylation and lower hepcidin expression. These parallel responses support an association between aerobic exercise and reduced IL-6/JAK2/STAT3–hepcidin signaling. They also provide a plausible explanation for the concurrent increase in FPN1 expression and reduction in hepatic Fe^2+^.

IL-6 responses to exercise are tissue- and time-dependent. Acute exercise can transiently increase circulating and skeletal-muscle-derived IL-6, whereas regular exercise training may reduce chronic low-grade inflammation and basal inflammatory signaling [22,23]. Therefore, the reduced hepatic IL-6 expression observed after eight weeks of training likely reflects attenuation of chronic hepatic inflammatory stress rather than the acute myokine response induced by a single exercise session.

However, the present data cannot confirm that the IL-6/JAK2/STAT3–hepcidin pathway mediated the protective effects of exercise. No IL-6-neutralizing intervention, JAK2/STAT3 inhibitor, hepcidin antagonist, or genetic model was used. Moreover, reductions in hepatic IL-6 signaling may have occurred secondary to the overall improvement in hepatic lipid accumulation and inflammatory stress rather than representing the initiating mechanism. The findings should therefore be interpreted as evidence of coordinated suppression of hepatic inflammatory and hepcidin-related signaling rather than proof that exercise improves MASLD specifically through this pathway.

### 4.3. Aerobic Exercise Attenuates Ferroptosis-Related Molecular Alterations

HFD feeding reduced GPX4 expression and hepatic GSH and SOD levels while increasing PTGS2 expression and MDA levels. This combination indicates impaired antioxidant defense and enhanced lipid peroxidation. GSH acts as an essential reducing co-substrate for GPX4-mediated detoxification of phospholipid hydroperoxides. GPX4 uses the reducing capacity of GSH to convert membrane phospholipid hydroperoxides into less reactive products, thereby limiting iron-dependent lipid peroxidation [24,25,26]. Reduced GPX4 expression or activity and depletion of GSH therefore increase cellular susceptibility to ferroptosis [24,25,26].

Exercise reversed these alterations to varying degrees. MEH and HEH produced stronger restoration of GPX4 expression and greater reductions in PTGS2 and MDA than LEH, while HEH generally produced the most consistent improvement in antioxidant-related indicators. Aerobic exercise has previously been shown to improve hepatic lipid metabolism and oxidative stress in experimental fatty liver models [17,27]. Clinical and preclinical evidence also indicates that regular exercise can reduce hepatic fat accumulation and improve metabolic and antioxidant homeostasis in MASLD or related metabolic conditions [28,29,30,31,32,33,34,35].

The increases in GPX4, GSH, and SOD and the reductions in PTGS2 and MDA were accompanied by improvements in liver histology, ALT, and AST. These findings suggest that enhanced antioxidant defense and reduced lipid peroxidation may contribute to the hepatoprotective effects of exercise. Previous studies in neurological disease models have similarly reported that exercise can increase GPX4 expression and attenuate iron accumulation or lipid peroxidation [36,37]. These studies provide indirect support for an exercise–ferroptosis relationship, although differences in tissue type and disease context limit direct extrapolation to MASLD.

Importantly, the present findings should be described as attenuation of ferroptosis-related alterations or reduced ferroptosis susceptibility, rather than direct inhibition of ferroptotic cell death. GPX4, PTGS2, MDA, GSH, and SOD are informative but are not individually specific for ferroptosis; PTGS2 can increase during inflammatory and oxidative responses independent of ferroptosis, and SOD mainly reflects general antioxidant capacity [23,30]. The study did not quantify lipid ROS with C11-BODIPY, assess characteristic mitochondrial ultrastructure by transmission electron microscopy, or test rescue with a ferroptosis inhibitor such as ferrostatin-1 or liproxstatin-1. ACSL4 and SLC7A11 were also not measured. Therefore, although the concordant changes across iron, antioxidant, and lipid-peroxidation indicators strengthen the biological interpretation, direct evidence of ferroptotic cell death remains absent.

The molecular analyses were based on only three independent biological replicates per group, and no formal a priori power calculation was performed. This limited sample may produce unstable effect estimates and reduces confidence in reproducibility. Future mechanistic studies should use an a priori sample-size calculation and increase molecular biological replicates, preferably to at least six animals per group when feasible.

### 4.4. Duration-Based Aerobic Exercise Load May Influence the Magnitude of Hepatoprotection

The three exercise protocols reached the same final treadmill speed, whereas the primary difference was the duration of each exercise session. Accordingly, the observed differences should be attributed mainly to duration-based exercise load and cumulative exercise volume rather than exercise intensity alone. HEH produced the most consistent improvements in metabolic parameters, hepatic steatosis, liver enzymes, Fe^2+^ accumulation, inflammatory signaling, and ferroptosis-related markers.

A longer exercise duration may increase cumulative energy expenditure, substrate utilization, and fatty acid oxidation, thereby reducing circulating lipid availability and hepatic lipid accumulation. Exercise is known to improve liver fat, insulin sensitivity, lipid metabolism, and liver function in MASLD and related fatty liver conditions [27,31,32,33,34,35]. Exercise-associated reductions in hepatic lipid accumulation may subsequently alleviate lipotoxicity, inflammatory signaling, oxidative stress, and iron-dependent lipid peroxidation [17,27,30]. The greater reductions in IL-6/JAK2/STAT3–hepcidin signaling and hepatic Fe^2+^ levels in the HEH group may therefore partly reflect the stronger overall metabolic improvement achieved by the longer-duration protocol.

However, the present study cannot determine why the HEH group showed the most consistent overall response. Daily food intake, exercise energy expenditure, and body composition or fat mass were not quantified. Thus, the superior outcomes in HEH may reflect greater cumulative exercise volume, a larger negative energy balance, altered appetite, reduced adiposity, or differences in exercise tolerance rather than session duration alone. Moreover, steatosis, inflammation, iron handling, and ferroptosis-related markers improved in parallel, so the present design cannot identify which change was the primary mediator and which changes were secondary consequences.

The findings should not be interpreted to mean that progressively longer or more demanding exercise necessarily produces greater benefit. Prolonged or strenuous exercise can increase lipid peroxidation and oxidative stress when the stimulus exceeds adaptive capacity [38,39]. Although the 90-min protocol was beneficial under the present rat experimental conditions, physiological stress indicators, corticosterone, fatigue, and exercise performance were not assessed. Crucially, the 90-min/day treadmill protocol represents a model-specific experimental exposure in rats; it is not equivalent to, and must not be directly converted into, a 90-min/day exercise prescription for humans. Translation to clinical exercise prescription requires consideration of relative exercise intensity, cardiorespiratory fitness, total weekly workload and energy expenditure, recovery capacity, and species-specific physiology. Future studies should compare a broader range of exercise volumes, quantify energy expenditure, food intake, body composition, and stress responses, and determine whether the dose-response relationship is linear or has an optimal adaptive window.

## 5. Conclusions and Practical Interpretation

Within the tested 30–90 min/day range, aerobic exercise was associated with progressively more consistent improvement in HFD-induced MASLD, with the 90-min protocol producing the broadest overall response across the full panel of outcomes. However, the absence of significant differences among exercise-duration groups for several body-weight and glucose-tolerance comparisons indicates that the response was not uniformly duration dependent. The mechanistic data support a coordinated association among reduced chronic IL-6/JAK2/STAT3-hepcidin signaling, improved hepatic iron handling, and a lower burden of lipid-peroxidation and ferroptosis-related molecular alterations, but they do not prove pathway mediation or inhibition of ferroptotic cell death. Importantly, the 90-min/day treadmill protocol is a model-specific experimental exposure in rats and must not be interpreted as, or directly converted into, a 90-min/day exercise recommendation for humans. Further dose-response studies using species-appropriate workload normalization, direct energy-balance measurements, adequately powered molecular analyses, and ferroptosis-specific interventions are required before clinical translation.

## 6. Study Limitations

This study has several limitations. First, only male SD rats were studied; sex- and species-related differences may limit extrapolation to female animals and humans. Second, qRT-PCR and Western blot analyses used only three independent biological replicates per group and no formal power calculation; these molecular findings are therefore exploratory. Third, the absence of an NFD-plus-exercise group prevents separation of disease-specific therapeutic responses from exercise effects that may also occur in healthy animals. Fourth, food intake, exercise energy expenditure, and body composition were not measured, so the greater overall response to HEH cannot be attributed solely to duration or cumulative exercise volume. Fifth, direct ferroptosis evidence—such as C11-BODIPY lipid-ROS measurements, transmission electron microscopy, or rescue with ferrostatin-1/liproxstatin-1—was not obtained. Finally, the 90-min rat treadmill protocol cannot be directly converted into a human exercise prescription. These limitations require cautious, associative interpretation of the proposed links among exercise load, inflammatory signaling, iron homeostasis, and ferroptosis-related alterations.

## Figures and Tables

**Table 1:** Exercise training program for rats.

**LEH Experimental period**	**Time**	**Speed** **(** **m/min)**	**Training duration (min)**
Week1	Day1	15	5
	Day2	16	10
	Day3	17	15
	Day4	18	20
	Day5	19	25
	Day6	20	30
Weeks 2–8	Days 1–6	20	30
**MEH Experimental period**	**Time**	**Speed (m/min)**	**Training duration (min)**
Week1	Day1	15	30
	Day2	16	40
	Day3	17	45
	Day4	18	50
	Day5	19	55
	Day6	20	60
Weeks 2–8	Days 1–6	20	60
**HEH Experimental period**	**Time**	**Speed (m/min** **)**	**Training duration (min)**
Week1	Day1	15	60
	Day2	16	70
	Day3	17	75
	Day4	18	80
	Day5	19	85
	Day6	20	90
Weeks 2–8	Days 1–6	20	90

**Table 2:** Primer sequences used for qRT-PCR.

Gene	Forward Primer Sequence (5′-3′)	Reverse Primer Sequence (5′-3′)
IL-6	AGTGGGCTAAGGACCAAGACC	TAGCACACTAGGTTTGCCGAG
FPN1	GTTGGCCAGATTATGACATTCG	ATTCCAACCAGAAATGAAACCA
DMT1	GCTGCTCCAAACTGTGAGCTA	ACGGTGACACACTTCAGCAA
Hepcidin	TGCCTGTCTCCTGCTTCTCCTC	AGAGCCGTAGTCTGTCTCGTCTG
GPX4	TACACTCAGCTAGTCGATCTGCAT	GCCGTTCTTATCAATGAGAAACTT
PTGS2	ACTCTATCACTGGCATCCG	GAGCAAGTCCGTGTTCAAG
β-actin	CTGAACGTGAAATTGTCCGAGA	TTGCCAATGGTGATGACCTG

## Data Availability

All data presented in this study are available within the article.

## Abbreviations

ALT	Alanine aminotransferase
ANOVA	Analysis of variance
AST	Aspartate aminotransferase
AUC	Area under the curve
BCA	Bicinchoninic acid
cDNA	Complementary deoxyribonucleic acid
DMT1	Divalent metal transporter 1
FPN1	Ferroportin 1
gDNA	Genomic deoxyribonucleic acid
GPX4	Glutathione peroxidase 4
GSH	Glutathione
H&E	Hematoxylin and eosin
HDL-C	High-density lipoprotein cholesterol
HEH	High-load aerobic exercise
HFD	High-fat diet
HRP	Horseradish peroxidase
IL-6	Interleukin-6
IR	Insulin resistance
ITT	Insulin tolerance test
JAK2	Janus kinase 2
LDL-C	Low-density lipoprotein cholesterol
LEH	Low-load aerobic exercise
MASH	Metabolic dysfunction-associated steatohepatitis
MASLD	Metabolic dysfunction-associated steatotic liver disease
MDA	Malondialdehyde
MEH	Moderate-load aerobic exercise
mRNA	Messenger ribonucleic acid
NAS	Nonalcoholic fatty liver disease activity score
NFD	Normal-fat diet
NIH	National Institutes of Health
OGTT	Oral glucose tolerance test
PBS	Phosphate-buffered saline
PCR	Polymerase chain reaction
p-JAK2	Phosphorylated Janus kinase 2
p-STAT3	Phosphorylated signal transducer and activator of transcription 3
PTGS2	Prostaglandin-endoperoxide synthase 2
PVDF	Polyvinylidene difluoride
qRT-PCR	Quantitative real-time polymerase chain reaction
RNA	Ribonucleic acid
ROS	Reactive oxygen species
SD	Sprague–Dawley
SDS-PAGE	Sodium dodecyl sulfate-polyacrylamide gel electrophoresis
SEM	Standard error of the mean
SOD	Superoxide dismutase
STAT3	Signal transducer and activator of transcription 3
TBST	Tris-buffered saline with Tween 20
TC	Total cholesterol
TG	Triglycerides
